# Electrochemical growth of Co nanowires in ultra-high aspect ratio InP membranes: FFT-impedance spectroscopy of the growth process and magnetic properties

**DOI:** 10.1186/1556-276X-9-316

**Published:** 2014-06-25

**Authors:** Mark-Daniel Gerngross, Jürgen Carstensen, Helmut Föll

**Affiliations:** 1 Institute for Materials Science, Christian-Albrechts-University of Kiel, Kaiserstrasse 2, Kiel 24143, Germany

**Keywords:** Co nanowires, single-crystalline InP membranes, FFT-impedance spectroscopy, Maxwell element, RC element

## Abstract

The electrochemical growth of Co nanowires in ultra-high aspect ratio InP membranes has been investigated by fast Fourier transform-impedance spectroscopy (FFT-IS) in the frequency range from 75 Hz to 18.5 kHz. The impedance data could be fitted very well using an electric circuit equivalent model with a series resistance connected in series to a simple *resistor-capacitor* (*RC*) element and a Maxwell element. Based on the impedance data, the Co deposition in ultra-high aspect ratio InP membranes can be divided into two different Co deposition processes. The corresponding share of each process on the overall Co deposition can be determined directly from the transfer resistances of the two processes. The impedance data clearly show the beneficial impact of boric acid on the Co deposition and also indicate a diffusion limitation of boric acid in ultra-high aspect ratio InP membranes. The grown Co nanowires are polycrystalline with a very small grain size. They show a narrow hysteresis loop with a preferential orientation of the easy magnetization direction along the long nanowire axis due to the arising shape anisotropy of the Co nanowires.

## Background

In this paper, the galvanic filling of InP membranes will be discussed which is an essential step for special magnetic field sensors based on magnetoelectric composites. Sensing biomagnetic signals either from the heart or the brain of a human have become more and more important in modern medical diagnostics, e.g. to detect malfunctions of the heart by magnetocardiography (MCG) [1,2] or to find the origin for seizures in the brain by magnetoencephalography (MEG) [3,4]. These biomagnetic signals to be detected lie in the order of 10^−12^ to 10^−15^ T. Up to now, this requires rather huge and expensive superconducting quantum interference device (SQUID)-based systems that limit the application to university hospitals or hospital centers. As an additional disadvantage, the SQUID-based systems cannot be applied directly to the patient because of the need for thermal insulation due to liquid helium respectively liquid nitrogen cooling of the SQUIDs. This gives rise to the potential replacement by magnetoelectric composite sensors. In principle, different composite geometries are possible. Magnetoelectric 1–3 composites - one-dimensional magnetostrictive structures in a three-dimensional piezoelectric matrix - have the potential advantage of millions of magnetoelectric elements in parallel and also the very high contact area between the magnetostrictive and piezoelectric component.

The galvanic deposition of magnetic and nonmagnetic metals into porous materials is a challenging field especially for ignoble metals, mainly in terms of conformal filling from the bottom of the pore [5-7]. Most of the deposition research has been done in porous alumina membranes [8-10]. It was recently shown in [11] that it is possible to galvanically grow dense Ni nanowires in ultra-high aspect ratio porous InP membranes when coating the pore walls with a very thin dielectric interlayer prior to the galvanic deposition. The dielectric layer electrically passivates the pore walls so that a nucleation of metal clusters on the pore walls is prevented. It is well known that the magnetic properties of galvanically grown nanowires strongly depend on the growth conditions. The galvanic deposition parameters have been widely exploited and optimized for thin films [12-18], but not for the application in high and ultra-high aspect ratio structures. The huge difference between thin films and high aspect ratio structures is the mass transport of the species taking part in the deposition reaction. In case of high aspect ratio structures, this mass transport is highly diffusion-limited. The present work makes use of the fast Fourier transform-impedance spectroscopy (FFT-IS) to characterize the growth process of Co nanowires directly at the metal electrolyte interface deep in the pore under specific deposition conditions. The obtained results are then correlated to the results of the structural and magnetic investigation of the Co nanowires/InP membrane composite.

## Methods

The templates for the growth of Co nanowires are porous InP membranes. These membranes are fabricated in an electrochemical multistep process. The porous InP membranes are fabricated from single-crystalline InP wafers sulfur-doped at a doping concentration of 1.1·10^17^ cm^−3^ and a resistivity of 0.019 Ωcm. The surface of the InP wafers is double-side polished and epi-ready. The wafer thickness is 400 ± 10 μm, and the sample size is *A* = 0.25 cm^2^. All electrochemical process steps are carried out in electrochemical double cell as described elsewhere [19].

The first step in the membrane formation is the electrochemical etching of the current-line-oriented pore (curro-pore) array. This is done in an aqueous 6 wt% HCl electrolyte at 20°C. To ensure a homogenous nucleation of the curro-pores, a voltage pulse of 17 V for 1 s is applied that is followed by a constant anodic potential of 10 V for 36 min for the growth of the curro-pores.

In the second step, the membrane is formed. This is done in a combined photoelectrochemical and photochemical process. At first, a layer consisting of crystallographically-oriented pores (crysto-pores) is grown in the bulk wafer back side that is subsequently dissolved photochemically. The etching is carried out in the same electrochemical cell in a 6 wt% aqueous HCl electrolyte at 20°C. More details on the fabrication process are given elsewhere [20].

In the third step, the membrane structure is post-etched in an HF/HNO_3_/EtOH/HAc (3:8:15:24) electrolyte at 20°C under a bias potential of −0.8 V for 48 h to obtain an overlapping of the space charge region (SCR) around each pore with SCRs around neighboring pores and therefore semi-insulating properties. Besides this effect, the post-etching also results in perfectly rectangular pores with pore walls exhibiting an equal thickness.

The final step of the template fabrication is the electric passivation of the pore walls by an 8-nm-thick layer of Al_2_O_3_ deposited by atomic layer deposition (ALD) to avoid unfavorable current flow through the pore walls during galvanic deposition. This is done in 80 cycles of trimethylaluminum (TMA) and H_2_O with extended diffusion time at 300°C in a Picosun Sunale R200 ALD tool (Espoo, Finland).

Prior to the galvanic Co deposition, a Au layer with a thickness of about 400 nm is deposited on the InP membrane back side serving as a plating base ensuring a complete coverage of the membrane back side.

The growth of Co nanowires in the InP membrane template is carried out in an electrochemical cell with a three-electrode configuration: a Pt counter electrode and a Pt pseudo-reference electrode, which are both immersed in the Co electrolyte. The Au plating base of the InP membrane template serves as the working electrode.

The Co electrolyte is an aqueous electrolyte with 60 g/l CoSO_4_ and 45 g/l H_3_BO_3_ adjusted to a pH value of 3 by HCl. The electrolyte is kept constantly at a temperature of 35°C. The Co nanowires are grown at a constant current density of 12 mA/cm^2^ for 20 min. During the entire deposition process, FFT-IS is performed, i.e. every 2 s, a spectrum of 26 frequencies from 75 Hz to 18.5 kHz is applied simultaneously and the corresponding impedance data is recorded as well as the deposition voltage. The impedance data are analyzed *ex situ.*

The InP membrane/Co nanowires composite structure was investigated using a ZEISS Supra 55 VP scanning electron microscope (SEM) (Oberkochen, Germany) and a Seifert X-ray diffraction (XRD) 3000 TT (Olympia, WA, USA) (Cu Kα = 0.154 nm). The magnetic properties were investigated by a Lake Shore 7300 vibrating sample magnetometer (VSM; Westerville, OH, USA).

## Results and discussion

### Impedance analysis of the galvanic Co nanowire growth

**Figure 1:**
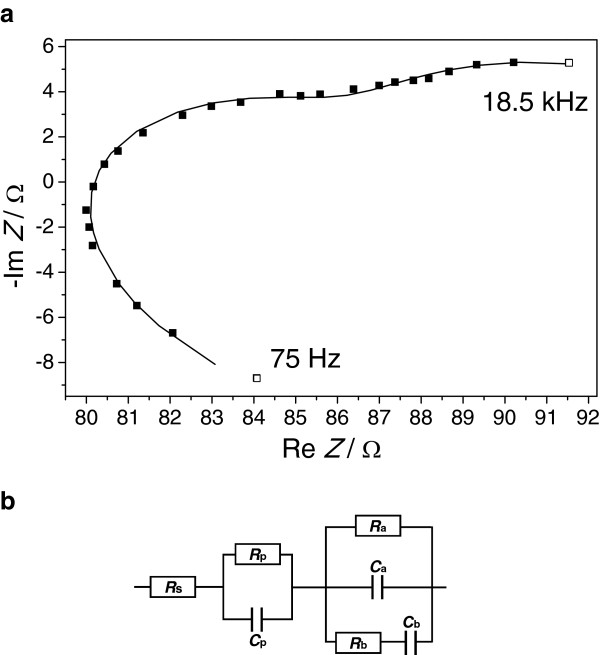
**Nyquist plot of the FFT-IS measurement and electric circuit representation of the Co deposition process. (a)** Typical Nyquist plot of the FFT-IS measurement during the galvanic growth of Co nanowires in InP membranes. The small boxes represent the measured data. The black line is the corresponding fit. **(b)** Corresponding equivalent circuit representation of the galvanic Co deposition process. The mathematical description is given in Equation 1.

The impedance data of the electrochemical growth of Co nanowires in an InP membrane were recorded as described in the ‘Methods’ section. Figure 1a shows the typical Nyquist plot obtained from the measured impedance data exhibiting three semicircles. The small boxes are the measured data points. The measured frequencies are indicated in the graph. The black line represents the fit.As one can see, the measured impedance data points and the fitting curve match very well. This shows the high quality and stability of the used fitting model. The electric equivalent circuit of the fit model is presented in Figure 1b with the corresponding mathematical description shown in Equation 1.

(1) $$Z_{\left(\omega \right)}=R_{S}+\frac{R_{p}}{1+\mathrm{i\omega}\tau_{p}}+\frac{1}{\frac{1}{R_{a}}+\mathrm{i\omega}C_{a}+\frac{\mathrm{i\omega}C_{b}}{1+\mathrm{i\omega}R_{b}C_{b}}}$$

It is a rather complex model consisting of a series resistor *R*_s_ that is connected in series with a *resistor-capacitor* (*RC*) element and in series with a Maxwell element. The *RC* element is a parallel arrangement of the resistor *R*_p_ and *C*_p_. The capacitor *C*_p_ itself does not occur as a separate fit parameter but is integrated in the time constant *τ*_p_. The Maxwell element is built up of a parallel arrangement of the resistor *R*_a_ and the capacitor *C*_a_ and the series connection of the resistor *R*_b_ and the capacitor *C*_b_. It is well known that the same impedance data can be described by several corresponding equivalent circuits. All possible arrangements starting from various Voigt model arrangements over Maxwell model arrangements have been evaluated. The chosen Maxwell model was the best-suited model to describe and explain the recorded impedance data most consistently for two reasons. The first reason is, it is shown in literature [15,17] that the Co deposition can occur via at least two reaction pathways. The second reason is that the decoupling of the seven fit parameters *vs.* time is best for the chosen Maxwell model in comparison to other investigated equivalent circuit models as will be discussed in the following.

**Figure 2:**
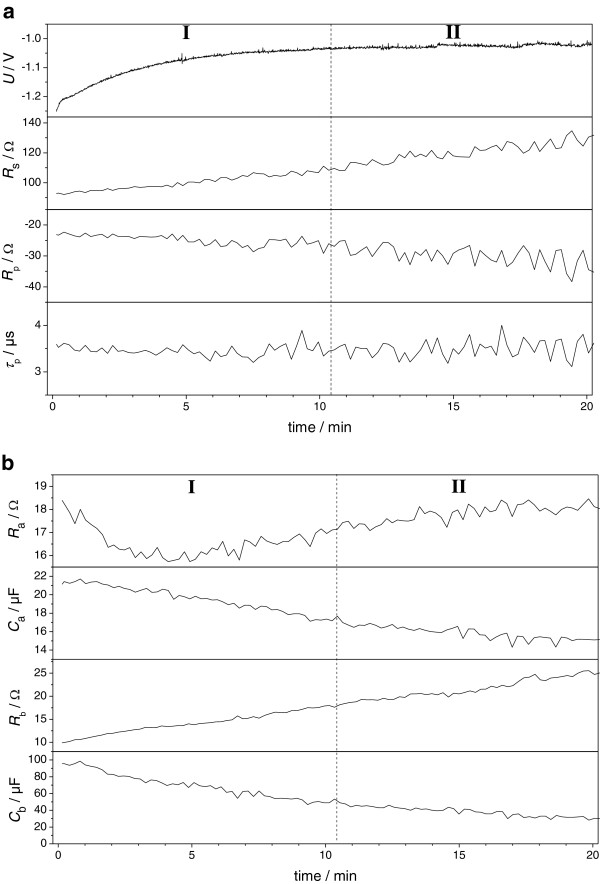
**The time dependence of the deposition voltage and the seven fit parameters. (a)** Deposition voltage *U* and the series resistance *R*_s_, transfer resistance *R*_p_, and the corresponding time constant *τ*_p_ and **(b)** the Maxwell element with *R*_a_, *C*_a_, *R*_b_, and *C*_b_ as a function of the deposition time at a constant current density of 12 mA/cm^2^.

The time dependence of the deposition voltage *U* and of the seven fit parameters - the series resistance *R*_s_, the transfer resistance *R*_p_, the corresponding time constant *τ*_p_ - are depicted in Figure 2a, the Maxwell resistances *R*_a_ and *R*_b_ and the corresponding capacities *C*_a_ and *C*_b_ in Figure 2b.

The Co deposition voltage *U* decreases exponentially with time starting from a value of about −1.25 V and reaches a constant deposition voltage of about −1 V after approximately 10.5 min. The series resistance *R*_s_ increases linearly with the time starting from about 90 Ω going up to about 130 Ω with slight oscillations towards the end.

The transfer resistance *R*_p_ is negative over the entire deposition time. It linearly increases starting from about −25 Ω up to about −35 Ω, reaching a constant level after about 16 min. Similar to the series resistance, also *R*_p_ shows oscillations towards the end but significantly more pronounced in amplitude. Unlike the *R*_p_, the associated process time constant *τ*_p_ remains constant over the entire deposition time. It also shows higher oscillations towards the end.

In the first three minutes, the Maxwell resistance *R*_a_ decreases linearly from about 18 Ω to about 16 Ω before *R*_a_ linearly increases to 18 Ω and saturates after 16 min with pronounced oscillations during the entire time. The associated capacity *C*_a_ does not exhibit the change in slope after three minutes as observed for *R*_a_. It decreases constantly from about 21 μF down to about 15 μF after 15 min before it saturates like *R*_a_.

The Maxwell resistance *R*_b_ increases linearly from about 10 Ω up to about 25 Ω. Compared to *R*_a_, the oscillations in *R*_b_ are extremely reduced. The corresponding capacity *C*_b_ decreases linearly from about 100 μF down to about 50 μF after 10.5 min and decreases further down to about 25 μF with a drastically reduced slope. Similar to *C*_a_, *C*_b_ only shows slight oscillations over the complete deposition time.

### Interpretation of the impedance fit parameters

As evident from the time dependence of the deposition voltage and the seven fit parameters shown in Figure 2, the galvanic Co deposition can be subdivided into two major sections. Section I is characterized by the exponential decline in the deposition voltage, section II by the constant deposition voltage.

The linear increase of *R*_s_ could be understood in terms of the Co nanowire growth. With proceeding deposition time, the Co nanowires increase their length contributing to the series resistence as well as, e.g. ohmic losses in the electrolyte.

A negative resistance can be understood as a process that is acting similar as a catalyst supporting the reaction. Hoare [21] found for Ni that boric acid in the deposition electrolyte acts in such a way that it is supporting the Ni deposition by forming complexes that can be reduced at lower overpotential compared to the boric acid-free electrolyte. Thus, the transfer resistance *R*_p_ and the process time constant *τ*_p_ could describe the influence of boric acid on the Co deposition in ultra-high aspect ratio InP pore arrays. The increase of *R*_p_ towards more negative values could be due to an increase in the concentration of boric acid in the pores with increasing deposition time as a result of a reduced diffusion limitation, since the Co nanowires grow towards the pore openings reducing the effective pore depth. The stronger oscillations in *R*_p_ might be due to a competition for adsorbing sites on the Co nanowire surface between boric acid-complexed Co ions and other adsorbed species.

The Maxwell resistance *R*_a_ could be related to the charge transfer resistance of the direct Co deposition. The decline in the first three minutes could be due to the diffusion limitation of the boric acid that forms complexes with Co^2+^ ions for an easier deposition. The following linear rise might be attributed to an increased surface coverage of the growing Co nanowires by adsorbed ions impeding the Co deposition. The constant level in *R*_a_ after 16 min coincides with the constant level in *R*_p_ suggesting that these adsorbed ions might be related to boric acid, such as e.g. B(OH)_4_^−^. The ending of the diffusion limitation for the boric acid might be the reason for the constant level in *R*_a_ after 16 min.

The Maxwell capacity *C*_a_ could be attributed to the corresponding double layer capacity of the direct Co deposition. The decline in *C*_a_ correlates with the concentration increase of boric acid species due to a reduced diffusion limitation (see time dependence of *R*_p_) and mirrors also the constant level after 16 min.

The Maxwell resistance *R*_b_ and the capacity *C*_b_ describe the slowest process during the Co deposition. It could be related to the indirect Co deposition via Co(OH)_2_ as experimentally observed by Santos et al. [18]. This process takes place in parallel to the direct Co deposition process. Therefore, *R*_b_ is assigned to the charge transfer resistance of the Co deposition process via Co(OH)_2_. The constant gain in *R*_b_ indicates that the Co deposition via this process becomes more and more unfavorable compared to the direct Co deposition process represented by *R*_a_. After around 10.5 min, the charge transfer resistances of *R*_a_ and *R*_b_ exhibit the same value. This allows splitting the entire Co deposition process into two sections. In section I, *R*_b_ is lower than *R*_a_. This means that the Co deposition occurs primarily via the indirect mechanism (via Co(OH)_2_). In section II, the situation is vice versa. The Co deposition occurs primarily via the direct mechanism. The share of the direct Co deposition out of the overall process is determined by 1 − *R*_a_ / (*R*_a_ + *R*_b_). Consequently, the share of the Co deposition via Co(OH)_2_ is given by 1 − *R*_b_ / (*R*_a_ + *R*_b_). The absence of strong oscillations in *R*_b_ also indicates that this process appears to be independent from the ending of the diffusion limitation of boric acid.

The capacitance *C*_b_ is assigned to the corresponding double layer capacity of the indirect Co deposition. The decline in *C*_b_ could be explained in the same way as for *C*_a_. The change in the slope of *C*_a_ after about 10.5 min is most probably related to the now preferential Co deposition via the direct deposition process.

As an additional side reaction of the Co deposition, hydrogen can form [16], but a process related to this hydrogen evolution during the Co deposition could not be identified in the recorded FFT-IS data within the investigated frequency range, most probably because it is a very slow process that is outside the investigated frequency range as it is found for the Ni deposition [22].

### Structural characterization

**Figure 3:**
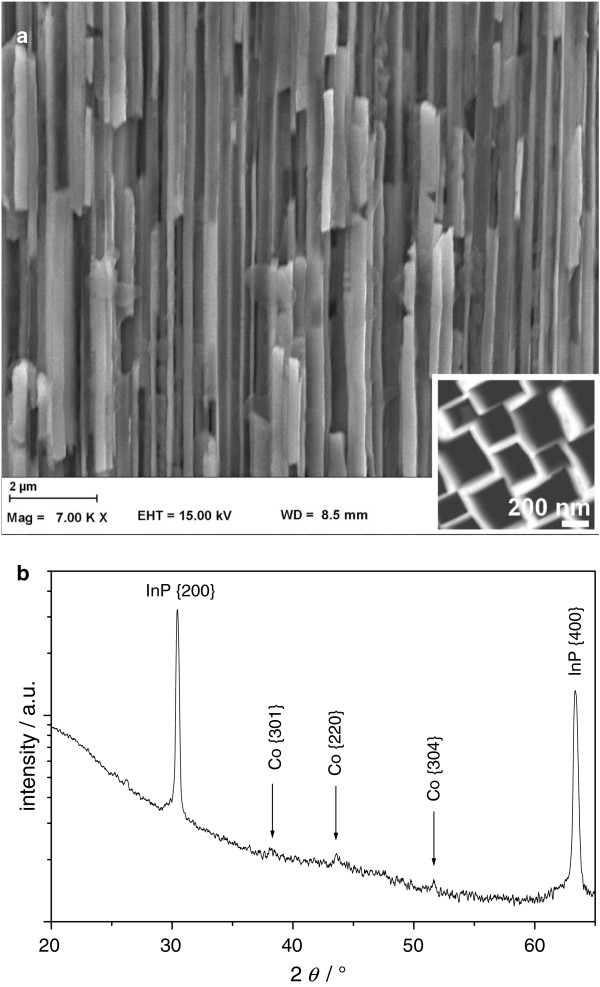
**SEM cross-sectional view and XRD pattern of the Co nanowire/InP membrane composite. (a)** SEM cross-sectional view on the Co nanowires/InP membrane composite; inset, SEM top view on the unfilled membrane. **(b)** XRD pattern of the Co nanowire/InP membrane composite.

The cross-sectional view on the Co nanowire/InP membrane is presented in Figure 3a. The Co nanowires appear brighter in the SEM image compared to the InP membrane. The fractures observed in the Co nanowires and the InP membrane are the result of the sample cleavage and are not a structure property. The Co nanowires grow from the Au plating base on the back side of the membrane. No nucleation of crystallites on the Al_2_O_3_-coated InP pore walls have been observed. The Co nanowires are dense and show no signs of porosity. They exhibit a rectangular shape since they grow in rectangular pores. The average nanowire diameter is about 300 nm, and the average distance between adjacent nanowires is about 60 nm.Figure 3b shows a typical XRD pattern of a Co nanowires/InP membrane composite. Two sharp peaks are found that are assigned to InP {200} and InP {400} as it is expected for single-crystalline (100) oriented InP wafers with pores along the [100] direction. The remaining three small and rather blurry peaks can be assigned to Co {301}, Co {220}, and Co {304}. The Co nanowires are crystalline and exhibit the typical hcp crystal structure, but there are no signs of a texturing of the Co nanowires. The shape of the two Co peaks indicates small coherently scattering areas and, thus, rather small Co grain sizes. Correlating this to the results of the FFT-IS suggests that the Co deposition via two simultaneously occurring processes favors the nucleation of new Co crystallites rather than the growth of already existing crystallites.

### Magnetic characterization

In general, it is decisive that the magnetization in the magnetic material is aligned perpendicular to the applied magnetic field for an optimal magnetostrictive effect, e.g., if the magnetization in the magnetic material is parallel to applied field, the magnetostrictive effect is zero. Another important factor for the application as magnetoelectric sensor is a small hysteresis loop, since magnetic AC fields shall be measured.

**Figure 4:**
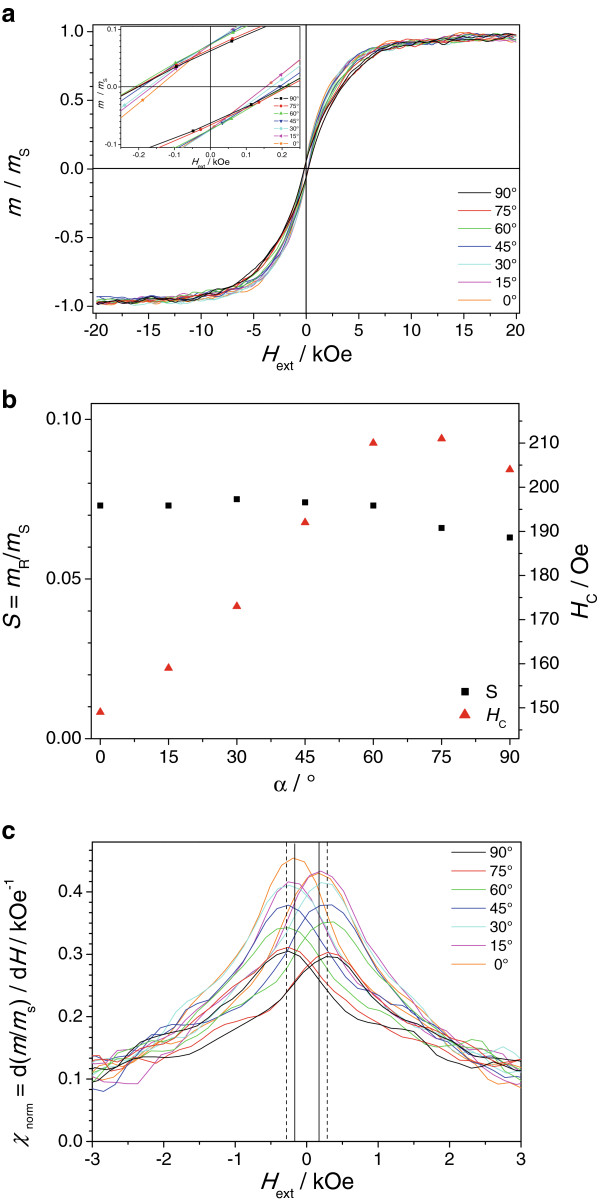
**Angular dependent hysteresis loops and magnetic properties of the Co nanowire/InP composite. (a)** Angular-dependent normalized hysteresis loops of the Co nanowires/InP membrane composite obtained by VSM measurement from *α* = 0° (*H* || *z*) to *α* = 90° (*H* ⊥ *z*); inset, high magnification of the hysteresis loops around *m*/*m*_s_ = 0. **(b)** Angular dependence of the remanence squareness *S* and the coercivity *H*_C_. **(c)** Angular dependence of the differential susceptibility of the Co nanowires/InP membrane obtained by VSM measurement at *α* = 0° (*H* || *z*) to *α* = 90° (*H* ⊥ *z*).

The magnetic properties of the Co nanowires/InP membrane composite are characterized by angular-dependent measurements of the hysteresis loops. The hysteresis loops are measured under various angles *α* between the external magnetic field *H* and the long nanowire axis *z* starting from *α* = 0° (*H* || *z*) to *α* = 90° (*H* ⊥ *z*). The detailed view of the axis intercepts are given in the inset of Figure 4a. The hysteresis loops are narrow and show a distinct, but not pronounced, angular dependence. With increasing angle *α*, a tilting of the hysteresis loops is observed. From these hysteresis loops, the remanence squareness *S*, the coercivity *H*_C_, and the differential normalized susceptibility *χ*_norm_ are extracted. The small oscillations in the hysteresis loops are measurement artifacts occurring at elevated sweep rates of the magnetic fields.

**Figure 5:**
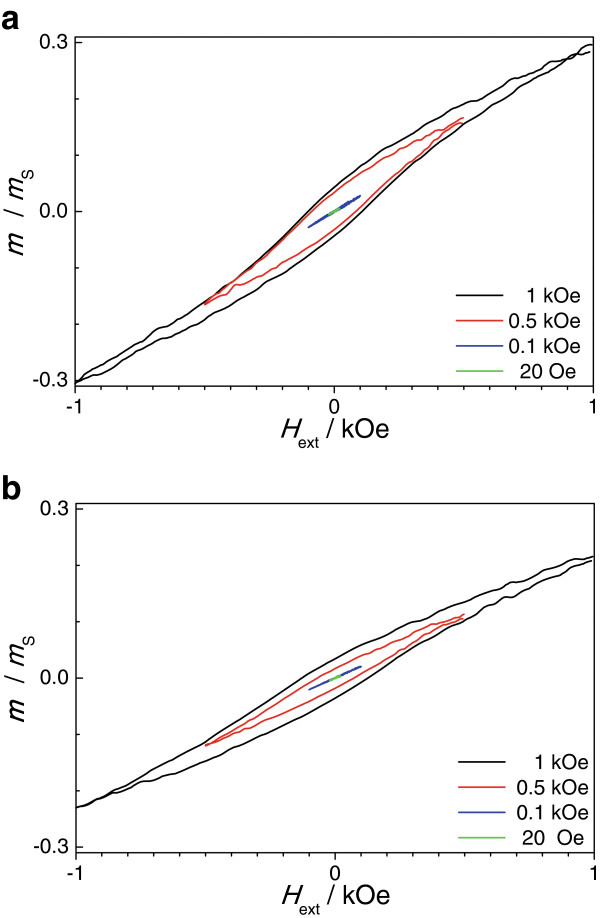
**Minor hysteresis loops of the Co nanowires/InP membrane composite. (a)** Minor hysteresis loop of the Co nanowires/InP membrane composite obtained by VSM measurement at *α* = 0° (*H* || *z*) and **(b)** at *α* = 90° (*H* ⊥ *z*).

The angular dependence of the remanence squareness is extracted from the measured hysteresis loops. It is depicted in Figure 4b. From *α* = 0° to *α* = 60°, the remanence squareness is rather constant with a value of around 0.07 and reduces slightly to about 0.06 with further increasing angle *α*. From these data, the easy magnetization direction of the Co nanowires cannot be clearly identified. Therefore, minor hysteresis loops with a field amplitude *H*_a_ between 20 Oe and 1 kOe are performed for *α* = 0° and *α* = 90° being shown in Figure 5a and b. The minor hysteresis loops for *α* = 0° and *α* = 90° show differences in the following three parameters, hysteresis loss and maximum normalized magnetization *m*_a_/*m*_s_ and the slope of the minor loops for very small *H*_a._

For *α* = 0°, the hysteresis losses of the 0.5 and 1 kOe minor loops are significantly higher compared to the corresponding minor loops for *α* = 90°. The same behavior is found for the maximum normalized magnetization. This behavior suggests that the easy magnetization direction of the Co nanowires lies along the long nanowire axis *z* (*α* = 0°) due to the high aspect ratio of the Co nanowires giving rise to a pronounced shape anisotropy that exceeds the magnetocrystalline anisotropy of Co [23]. The remanence squareness of 0.07 found for the easy magnetization direction is very low compared to a single nanowire with the magnetization also along the long nanowire axis *z*[24]. One could understand this behavior by taking into account the nucleation of domains with inverse magnetization at the bottom or at the top of the Co nanowires. These domains with inverse magnetization could efficiently reduce stray fields and might be also the reason for the reduced the remanence squareness. The magnetostatic interactions between neighboring Co nanowires might also play an important role, since the interwire distance is far smaller compared to the diameter of the Co nanowires.

Another interesting effect is that for external magnetic fields *H*_a_ larger than 500 Oe, the minor loops show a distinct hysteresis that disappears completely for very small *H*_a_ (20 and 100 Oe). These minor loops show a reversible linear magnetic field dependence with a higher slope observed for *α* = 0°. The reversible linear magnetic field dependence means that the magnetization reversal at very small fields *H*_a_ occurs by domain rotation and reversible domain wall motion and not by irreversible domain wall motion as observed for higher external fields.

The angular dependence of the coercivity is presented in Figure 4b. The coercivity shows a completely different angular behavior. It is smallest for *α* = 0° (around 150 Oe) and increases constantly to about 210 Oe for *α* = 60°, where it peaks for *α* = 60° and *α* = 75° before it slightly decreases to around 205 Oe for *α* = 90°. The magnified view on the differential normalized susceptibility *χ*_norm_ around *H* = 0 Oe - depicted in Figure 4c - shows an inverse angular behavior with respect to the maximum *χ*_norm_. With increasing angle *α*, the maximum *χ*_norm_ decreases steadily from about 0.43/kOe for *α* = 0° reaching a plateau at about 0.3/kOe for *α* = 75° and *α* = 90°. In addition to that, two characteristic peak positions are observed represented by the two solid lines at around 160 Oe and by the two dashed lines at around 280 Oe.

These unusual experimental results in the angular dependence of *H*_C_ and *χ*_norm_ could be consistently described taking into account the magnetization reversal that does not occur by a single magnetization reversal mechanism but by two different magnetization reversal mechanisms depending on the incidence angle α of the external magnetic field. The peak positions of *χ*_norm_ suggest that magnetization reversal mechanism I is predominant for *α* = 0° and becomes less dominant with increasing *α*, while the dominance of mechanism II increases with increasing *α*. Therefore, the maximum in *H*_C_ for *α* = 60° and *α* = 75° could be understood as the result of an interplay between the two magnetization reversal modes. The exact type of these magnetization reversal mechanisms could not be identified by the conducted hysteresis loop measurements. Nevertheless, one might speculate that these reversal modes are most probably the transversal and vortex magnetization reversal mode as found by micromagnetic simulations for Ni nanowires by Han et al. [25].

Correlating these magnetic results with the structural characterization, one could understand the comparatively high coercivity of the Co nanowires as a direct consequence of the small grain size accompanied by the high amount of grain boundaries that hinder the domain wall movement. The small grain size itself is most probably a consequence of the deposition via the two simultaneously occurring Co deposition processes, as already discussed in the first part of this paper.

## Conclusions

The electrochemical growth of Co nanowires in ultra-high aspect ratio InP membranes could be successfully characterized by the analysis of the FFT-IS data. The corresponding fit model is represented by a rather complex electric equivalent circuit containing a series resistance and three RC elements. This fit model is not limited to the Co deposition but has also been successfully applied for the deposition of Ni in ultra-high aspect ratio InP membranes.

Based on the impedance data, the Co nanowire growth could be divided into two separate processes, most possibly the direct Co deposition and the indirect Co deposition via Co(OH)_2_. The share of each Co deposition process on the overall Co deposition can be determined directly from the transfer resistances of the two processes obtained from the fitted impedance data. These also indicate a beneficial effect of boric acid on the Co deposition. This characterization of the Co deposition process by FFT-IS will help in optimizing the deposition parameters such as temperature, deposition current, electrolyte composition, etc. with respect to the crystal orientation and thus also of the magnetic properties necessary for the application in magnetoelectric 1– 3 composites.

## Competing interests

The authors declare that they have no competing interests.

## Authors’ contributions

MDG performed all experiments. All authors discussed the data and prepared the manuscript. All authors read and approved the final manuscript.
